# Usefulness of Age-Stratified N-Terminal Prohormone of Brain Natriuretic Peptide for Diagnosing Kawasaki Disease

**DOI:** 10.1155/2017/6263121

**Published:** 2017-11-22

**Authors:** Sang Hoon Lee, Eun Song Song, Somy Yoon, Seunghee Hong, Hwa Jin Cho, Eun Mi Yang, Gwang Hyeon Eom, Gaeun Kang, Young Kuk Cho

**Affiliations:** ^1^Department of Pediatrics, Chonnam National University Medical School, Chonnam National University Hospital, Gwangju 61469, Republic of Korea; ^2^Department of Pharmacology, Chonnam National University Medical School, Gwangju 61469, Republic of Korea; ^3^Division of Clinical Pharmacology, Chonnam National University Hospital, Gwangju 61469, Republic of Korea

## Abstract

N-terminal prohormone of brain natriuretic peptide (NT-proBNP) was recently reported as a biomarker for diagnosing Kawasaki disease (KD). The basal NT-proBNP level, however, gradually decreases with age. We investigated the usefulness of an age-stratified cutoff value of NT-proBNP for diagnosing KD. All the patients enrolled in this study visited Chonnam National University Hospital between December 2007 and March 2016. The KD groups consisted of 214 patients with complete KD and 129 patients with incomplete KD. The control group included 62 children with simple febrile illness but without heart disease. Laboratory data including NT-proBNP level were evaluated. Each group was divided into subgroups according to patient age (<6 months, 6–12 months, 12–24 months, and >24 months), and different cutoff values of NT-proBNP were calculated. The cutoff values of NT-proBNP used to diagnose total KD and incomplete KD were 762 and 762 pg/mL (<6 months), 310 and 310 pg/mL (6–12 months), 326 and 326 pg/mL (12–24 months), and 208 and 137 pg/mL (>24 months), respectively. In conclusion, age-stratified NT-proBNP is a useful biomarker for the differential diagnosis of KD in patients with a simple febrile illness.

## 1. Introduction

Kawasaki disease (KD) is a systemic vasculitis that usually affects children and infants < 5 years of age [1, 2]. KD is thought to be the most common cause of acquired heart disease [3]. Among untreated patients or those subject to a treatment delay, 20–25% suffers from complications of coronary artery disease [3, 4]. Therefore, a prompt and accurate diagnosis and timely treatment of KD with intravenous immunoglobulin (IVIg) are necessary to minimize the complications associated with KD [3].

Because KD can only be diagnosed based on clinical symptoms, the diagnosis and treatment of KD patients are sometimes delayed; this is especially true for patients with incomplete KD, which is characterized by an incomplete presentation of diagnostic clinical symptoms [3]. Therefore, in cases of vague clinical symptoms, the diagnosis inevitably depends on adjuvant diagnostic markers. Some that are already broadly adopted are elevated C-reactive protein (CRP), elevated erythrocyte sedimentation rate (ESR), low albumin, anemia, elevation of alanine aminotransferase (ALT), a high platelet count after 7 days, and a high white blood cell count or pyuria [5].

N-terminal prohormone of brain natriuretic peptide (NT-proBNP) is an adjuvant marker used to diagnose KD, and many studies have illustrated its usefulness [6, 7]. Lee et al. reported that the cutoff value of NT-proBNP for diagnosing incomplete KD was 158 pg/dL, with 81% sensitivity and 74% specificity [6]. Another study reported that the cutoff value of NT-proBNP used to diagnose KD was 260 pg/dL, which displayed 93% sensitivity and 88% specificity [7]. However, the normal range of NT-proBNP varies widely with age [8]. The levels of NT-proBNP were the highest in the first few days of life, showed a marked decline in the first week, and continued to decline gradually with age [8]. Because of the natural age-based differences, applying the same cutoff value of NT-proBNP to each patient regardless of age would be unreasonable. Here, we investigated NT-proBNP as a biomarker by age in patients with KD or simple febrile illness and checked the usefulness of age-stratified cutoff values of NT-proBNP for diagnosing KD.

## 2. Methods

### 2.1. Patients

The two KD groups (incomplete and complete KD) included a total of 343 children who were diagnosed with KD and treated with IVIg at Chonnam National University Hospital between December 2007 and March 2016. Complete KD was diagnosed according to American Heart Association criteria suggested in 2017 [5]. The criteria include fever persisting for at least 5 days and fulfillment of at least four of five clinical symptoms [5]. In contrast, incomplete KD was diagnosed when a patient presented three or fewer symptoms and other possible causes of the fever were excluded [5]. The control group included 62 age-matched children who visited our center for an evaluation of febrile illness and underwent laboratory testing for NT-proBNP; we excluded those with heart diseases such as congenital heart disease, cardiomyopathy, and acute myocarditis, which can affect NT-proBNP levels [9]. Serum samples were obtained to measure serum NT-proBNP levels on the day that IVIg was started in patients with complete and incomplete KD and on the first day of admission in the control group. Serum NT-proBNP was analyzed using an electrochemiluminescence immunoassay (ElecsysProBNP Sandwich Immunoassay; Roche Diagnostics, Basel, Switzerland). At the same time, complete blood cell count (CBC); ESR; and levels of CRP, total protein, albumin, blood urea nitrogen (BUN), creatinine, aspartate aminotransferase (AST), ALT, creatine kinase (CK), CK-MB, myoglobin, and troponin-I were also obtained and analyzed. The reference values of NT-proBNP suggested by age were highest at birth and then significantly decreased over the first 2 years, especially before the age of 1 year. It showed little change with age after 2 years of age [8]. Therefore, we classified patients with KD and control patients into four subgroups by age considering the nature of these normal changes with age of NT-proBNP (<6 months, 6–12 months, 12–24 months, and >24 months).

### 2.2. Statistics

SPSS (version 23.0; SPSS, Chicago, IL, USA) was used for the data analyses. Continuous variables are presented as means ± standard deviations. The chi-square test was used to assess the statistical significance of differences between the independent variables. To compare data among more than three groups, analysis of variance (ANOVA) was employed, followed by Tukey's honestly significant difference post hoc test. When the homogeneity of variance was not present according to Levene's test, Dunnett's T3 was used for the post hoc test. To evaluate the correlation between two serologic factors, both Pearson's test and Spearman's rho test were utilized. The KD groups were evaluated by multinomial logistic regression including NT-proBNP, CRP, platelet count, and albumin as fixed effects. When the normal distribution was not assumed on the Shapiro-Wilk test, the Kruskal-Wallis test followed by the Bonferroni correction was used. The cutoff values for diagnosing KD were obtained using receiver operating characteristic (ROC) curve analysis. *P* values < 0.05 were considered statistically significant.

### 2.3. Ethics Statement

The purpose of this study was explained in detail to each patient's parent, who provided informed consent after careful consideration. Approval for the present study was obtained from the Institutional Review Board of Chonnam National University Hospital (protocol number I-2009-09-103). All data were treated confidentially.

## 3. Results

### 3.1. Patient Characteristics

Patient demographics are shown in Table 1. The total number of KD patients was 343, which included complete KD patients (*n* = 214, 62.4%) and incomplete KD patients (*n* = 129, 37.6%). In the complete KD group, the male-to-female ratio was 1.53 : 1 (136 versus 78) and the mean patient age was 2.8 ± 1.8 years. The complete KD group was classified into four subgroups by age (<6 months [*n* = 21, 9.8%], 6–12 months [*n* = 20, 9.3%], 12–24 months [*n* = 39, 18.2%], and >24 months [*n* = 134, 62.6%]) (Table 2). In the incomplete KD group, the male-to-female ratio was 1.39 : 1 (75 versus 54) and the mean patient age was 2.3 ± 2.0 years. The incomplete KD group was divided into four subgroups (<6 months [*n* = 25, 19.4%], 6–12 months [*n* = 22, 17.1%], 12–24 months [*n* = 21, 16.3%], and >24 months [*n* = 61, 47.3%]) (Table 2). Sixty-two age-matched febrile illness patients were enrolled as controls; the male-to-female ratio was 3.13 : 1 (47 versus 15) and the mean age was 2.9 ± 2.7 years. Neither sex nor fever duration differed significantly among the three groups. There were no significant differences in measurement time of NT-proBNP in patients with complete KD (4.2 ± 1.9 days), patients with incomplete KD (4.5 ± 2.5 days), and simple febrile illness controls (4.8 ± 3.9 days, *P* = 0.133). The clinical diagnoses in the control group were as follows: pneumonia (*n* = 19), acute tonsillitis (*n* = 15), cervical lymphadenitis (*n* = 9), acute bronchiolitis (*n* = 6), urinary tract infection (*n* = 5), acute cellulitis (*n* = 3), acute epiglottitis (*n* = 2), bacteremia (*n* = 2), and bacterial keratoconjunctivitis (*n* = 1). Nine patients yielded positive bacterial blood cultures. The control group was classified into four subgroups based on age (<6 months [*n* = 8, 12.9%], 6–12 months [*n* = 11, 17.7%], 12–24 months [*n* = 12, 19.4%], and >24 months [*n* = 31, 50.0%]) (Table 2).

### 3.2. Laboratory Findings at Diagnosis in Patients with KD and Simple Febrile Illness

**Figure 1:**
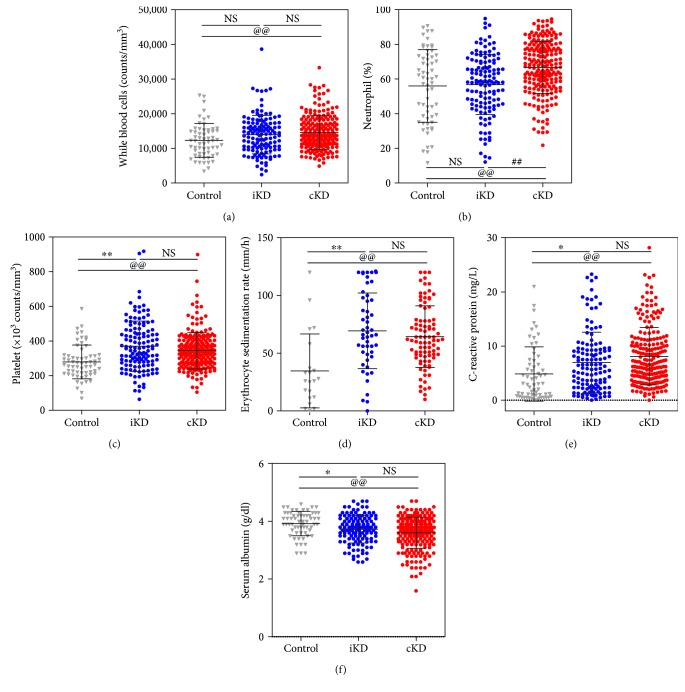
Dot plots of white blood cells (a), neutrophil percentage (b), platelet count (c), erythrocyte sedimentation rate (d), C-reactive protein level (e), and serum albumin level (f) in patients with complete Kawasaki disease (cKD), incomplete KD (iKD), and febrile illness (control). ^∗^*P* < 0.05. ^∗∗^, ^@@^, and ^##^*P* < 0.01. NS: not significant.

Laboratory data from the complete and incomplete KD groups and the febrile control group at the time of diagnosis are shown in Figure 1 and Tables 1 and 2. At the time of diagnosis, the white blood cell count in complete KD patients (14,561 ± 4855/mm^3^) was significantly higher than that in febrile illness controls (12,268 ± 4753/mm^3^, *P* = 0.007) (Figure 1(a)). The percentage of neutrophils in complete KD patients (66.3 ± 15.7%) was significantly higher than those in incomplete KD patients (56.8 ± 17.3%, *P* < 0.001) and febrile illness controls (56.0 ± 21.0%, *P* = 0.002) (Figure 1(b)). The platelet count in both complete KD patients (343 ± 104 × 10^3^/mm^3^) and incomplete KD patients (370 ± 145 × 10^3^/mm^3^) was significantly higher than that in controls (279 ± 97 × 10^3^/mm^3^, *P* < 0.001 for both) (Figure 1(c)). The ESR in complete KD patients (64.3 ± 26.6 mm/h) and in incomplete KD patients (69.3 ± 32.8 mm/h) was also significantly higher than that in controls (34.7 ± 32.0 mm/h, *P* < 0.001 for both) (Figure 1(d)). The CRP level in complete KD patients (8.1 ± 5.3 mg/dL) and incomplete KD patients (7.0 ± 5.6 mg/dL) was also significantly higher than that in febrile illness controls (4.8 ± 5.0 mg/dL, *P* < 0.001 and *P* = 0.039, resp.) (Figure 1(e)). Serum albumin levels in complete KD patients (3.6 ± 0.6 g/dL) and incomplete KD patients (3.7 ± 0.5 g/dL) were significantly lower than that in controls (3.9 ± 0.4 g/dL, *P* < 0.001 and *P* = 0.045, resp.) (Figure 1(f)). Although we found significant alterations in several laboratory markers in complete or incomplete KD, as mentioned above, there were no statistically significant differences between complete KD and incomplete KD patients' WBC counts, platelet counts, ESR, CRP, or serum albumin levels.

### 3.3. NT-proBNP Levels in Patients with KD and Simple Febrile Illness

**Figure 2:**
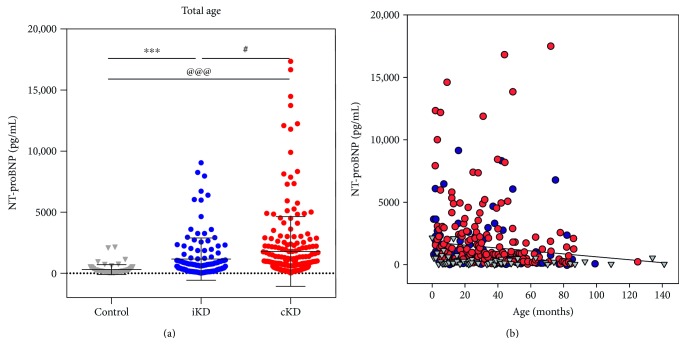
Dot plots of N-terminal prohormone of brain natriuretic peptide (NT-proBNP) in patients with complete Kawasaki disease (cKD), incomplete KD (iKD), and febrile illness (control) (a). NT-proBNP values by age (b). Black solid line depicts linear regression. ^#^*P* < 0.05. ^∗∗∗^ and ^@@@^*P* < 0.001.

In addition to the common serum chemical studies, we evaluated alterations in NT-proBNP levels. In all ages combined, the mean NT-proBNP levels in the complete KD group (1785 ± 2858 pg/mL) and incomplete KD group (1153 ± 1725 pg/mL) were significantly higher than that in the control group (311 ± 400 pg/mL, *P* < 0.001 for both) (Table 1, Figure 2(a)). The mean NT-proBNP level in the complete KD group was higher than that in the incomplete KD group (*P* = 0.033). We further investigated whether the aberrant elevation of NT-proBNP was tightly linked with inflammatory biomarkers such as CRP, platelet count, and elevated liver enzymes. To adjust for confounding factors, we performed a logistic regression between the clinical markers as well as NT-proBNP in the KD groups (Table 3). There were no statistically significant interactions between NT-proBNP, CRP, platelet count, and serum albumin. NT-proBNP was an independent predictor of KD despite the adjustment for CRP, platelet, and albumin levels (*P* < 0.001). Therefore, a high NT-proBNP level instead of a high CRP level increases the likelihood of a diagnosis of KD.

### 3.4. NT-proBNP Level by Age

**Figure 3:**
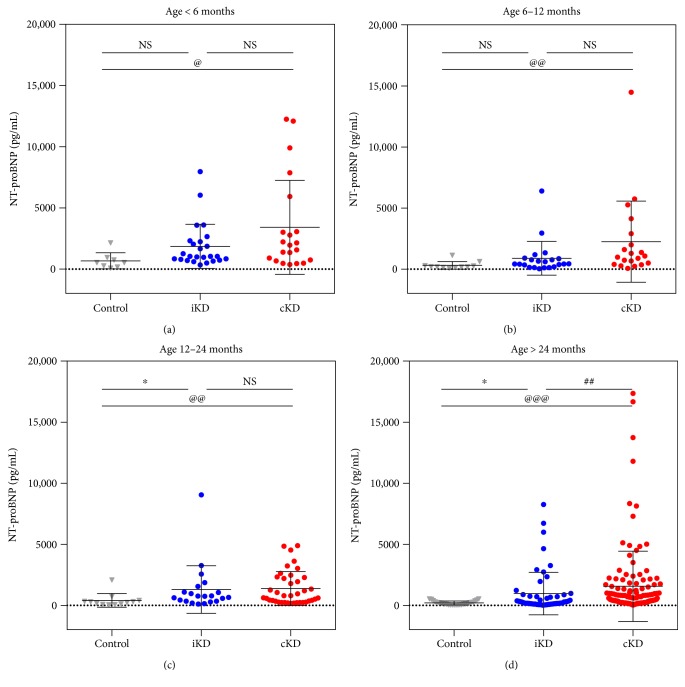
Dot plot of N-terminal prohormone of brain natriuretic peptide (NT-proBNP) in patients with complete Kawasaki disease (KD), incomplete KD, and febrile illness (control) according to age. ^∗^ and ^@^*P* < 0.05. ^@@^ and ^##^*P* < 0.01. ^@@@^*P* < 0.001. NS: not significant.

It has been reported that serum NT-proBNP is negatively correlated with a child's age [8]. Thus, we tested whether the sample distribution was closely linked with age using a bivariate correlation test. The serum NT-proBNP level tended to decrease as age increased (Pearson's correlation, *P* = 0.015; Spearman's rho, *P* < 0.001) (Figure 2(b)). Hence, we divided the patients into four groups to account for the effect of age and performed specific comparisons of the groups (<6 months, 6–12 months, 12–24 months, and >24 months). In the subgroups, the mean NT-proBNP level of patients with complete KD was significantly greater than that of age-matched controls. The specific distribution (Figure 3) and descriptive statistics (Table 2) are provided.

In patients < 6 months of age, the mean NT-proBNP level in the complete KD group (3404 ± 3844 pg/mL) was significantly higher than that in the control group (669 ± 660 pg/mL, *P* = 0.011) (Table 2, Figure 3(a)). Next, in patients 6–12 months of age, the mean NT-proBNP level in the complete KD group (2241 ± 3322 pg/mL) was significantly higher than that in the control group (301 ± 310 pg/mL, *P* = 0.002; Table 2, Figure 3(b)). In patients 12–24 months of age, the mean NT-proBNP levels in the complete KD group (1372 ± 1387 pg/mL) and incomplete KD group (1297 ± 1951 pg/mL) were significantly higher than that in the control group (398 ± 560 pg/mL, *P* = 0.004 and *P* = 0.024, resp.) (Table 2, Figure 3(c)). Finally, in the group > 24 months of age, the mean NT-proBNP levels in the complete KD group (1583 ± 2863 pg/mL) and the incomplete KD group (916 ± 1675 pg/mL) were significantly higher than that in the control group (188 ± 154 pg/mL, *P* < 0.001 and *P* = 0.020, resp.) (Table 2, Figure 3(d)). The mean NT-proBNP level in the complete KD group was also higher than that in the incomplete KD group (*P* = 0.001).

**Figure 4:**
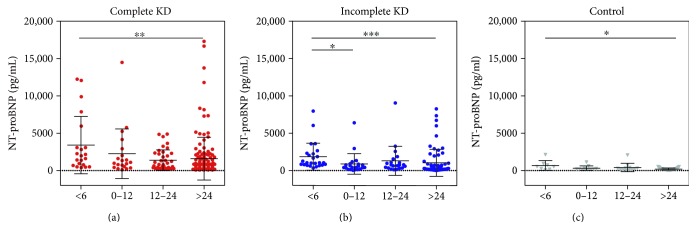
Dot plot of N-terminal prohormone of brain natriuretic peptide (NT-proBNP) in different age groups in patients with complete Kawasaki disease (KD) (a), incomplete KD (b), and febrile illness (control) (c). ^∗^*P* < 0.05. ^∗∗^*P* < 0.01. ^∗∗∗^*P* < 0.001. Otherwise, not significant.

Based on Pearson's correlation coefficient, we next postulated that the NT-proBNP level in younger patients with KD was also higher than that in older KD patients. The mean NT-proBNP level in the complete KD group < 6 months of age was higher than that in the group > 24 months of age (*P* = 0.002; Figure 4(a)). The mean NT-proBNP level in the incomplete KD group < 6 months of age was higher than those in the groups 6–12 months of age and >24 months of age (*P* = 0.018 and *P* < 0.001, resp.; Figure 4(b)). The mean NT-proBNP level in the control group < 6 months was higher than that in the group > 24 months of age (*P* = 0.041; Figure 4(c)).

### 3.5. Cutoff Value of NT-proBNP for Diagnosing KD

Because the NT-proBNP level decreases with age, there is a need for different cutoff values to diagnose KD by patient age; thus, we used ROC curves to determine these values (Table 4). We found the cutoff value of NT-proBNP in patients with complete KD, patients with incomplete KD, and all KD patients compared with the simple febrile illness control group.

The cutoff value in the total KD patient group of all ages was 289 pg/mL, with 71.7% sensitivity and 71.9% specificity (area under the curve (AUC) = 0.774). The cutoff value in the group aged < 6 months was 762 pg/mL, with 73.9% sensitivity and 75.0% specificity (AUC = 0.813). Similarly, the cutoff values in the other age groups (6–12 months, 12–24 months, and >24 months) were 310 pg/mL (sensitivity, 78.6%; specificity, 81.8%; AUC = 0.779), 326 pg/mL (sensitivity, 76.7%; specificity, 75.0%; AUC = 0.796), and 208 pg/mL (sensitivity, 70.8%; specificity, 69.7%; AUC = 0.782), respectively. Cutoff values were also obtained in the complete KD patient group. The cutoff value in the complete KD group of all ages was 326 pg/mL, with 73.8% sensitivity and 74.2% specificity (AUC = 0.795), while that in the group aged < 6 months was 821 pg/mL, with 71.4% sensitivity and 75.0% specificity (AUC = 0.810). The cutoff values in the other age groups (6–12 months, 12–24 months, and >24 months) were 315 pg/mL (sensitivity, 85.0%; specificity, 81.8%; AUC = 0.868), 330 pg/mL (sensitivity, 74.4%; specificity, 75.0%; AUC = 0.797), and 255 pg/mL (sensitivity, 73.1%; specificity, 77.4%; AUC = 0.836), respectively. Similar cutoff values were obtained in the incomplete KD patient group. The cutoff value in the incomplete KD group of all ages was 261 pg/mL, with 68.8% sensitivity and 67.2% specificity (AUC = 0.719), and the cutoff value in the group aged < 6 months was 762 pg/mL, with 76.0% sensitivity and 75.0% specificity (AUC = 0.815). The cutoff values in the other age groups (6–12 months, 12–24 months, and >24 months) were 310 pg/mL (sensitivity, 72.7%; specificity, 81.8%; AUC = 0.698), 326 pg/mL (sensitivity, 81.0%; specificity, 75.0%; AUC = 0.794), and 137 pg/mL (sensitivity, 70.5%; specificity, 54.5%; AUC = 0.665), respectively.

## 4. Discussion

Because a definitive diagnostic test for KD does not exist, diagnosis depends on a combination of clinical and laboratory findings [10]. Therefore, diagnosing KD is challenging in patients who show nonspecific symptoms or do not fulfill all of the criteria [11]. For this reason, many adjuvant diagnostic markers have been proposed, such as cytokines like helper T-cell (Th) 1, Th 2, interleukin- (IL-) 6, IL-20, tumor necrosis factor- (TNF-) alpha, and NT-proBNP [9].

Pre-proBNP, an analog of NT-proBNP, consists of 134 amino acids and is transformed into proBNP (108 amino acids), which is released from cardiac myocytes into the bloodstream. It is then broken down into bioactive BNP (32 amino acids) and NT-proBNP (76 amino acids). Usually, NT-proBNP plays no function in the body, but it can be used to indirectly measure the level of bioactive BNP [6]. Because of its stability and longer half-life, NT-proBNP is easier to use than bioactive BNP [7, 12]. Bioactive BNP and NT-proBNP have already been used to predict patient prognosis in adults with left ventricular dysfunction, ischemic heart disease, and hypertrophic cardiomyopathy [13]. Also, because it is positively related to the severity of congestive heart failure and left ventricular dysfunction, NT-proBNP is a useful biomarker in patients with cardiac disease [13].

Here, we found that the mean NT-proBNP in complete and incomplete KD patients was significantly higher than that in simple febrile illness patients in all age-stratified subgroups. Also, the mean NT-proBNP in complete KD patients was higher than that in incomplete patients. Our data revealed that the cutoff values for diagnosing KD and incomplete KD were 289 pg/mL (71.7% sensitivity and 71.0% specificity) and 261 pg/mL (72.1% sensitivity and 74.2% specificity), respectively. Many previous studies suggested cutoff values for distinguishing KD or incomplete KD from simple febrile illness [6, 7, 14]. Cho et al. recently reported that NT-proBNP might be a more useful marker than high-sensitivity CRP (hs-CRP) for diagnosing KD [14]. Those authors found that NT-proBNP levels were significantly higher in 59 KD patients (749.66 ± 997.11 pg/mL) than in 45 febrile illness patients used as age-matched controls (174.41 ± 144.30 pg/mL, *P* < 0.001) and that the differential diagnostic cutoff value of NT-proBNP was 235.2 pg/mL, with a sensitivity of 66.10% and a specificity of 77.08%. We also found limitations of using CRP to diagnose KD. Although an elevated CRP level is widely accepted as a common laboratory finding in KD patients, we found that an increase in CRP is not an independent marker but, rather, a confounder on logistic regression analysis. On the contrary, NT-proBNP itself was a powerful diagnostic marker of KD and useful in distinguishing it from simple febrile illness.

Lee et al. also investigated the cutoff level of NT proBNP that allowed them to distinguish 33 incomplete KD patients from 19 simple febrile illness patients. They proposed that an NT-proBNP cutoff value of 158 pg/mL provided a sensitivity of 81% and a specificity of 74% for the diagnosis of incomplete KD [6]. Another study reported that the cutoff value of NT-proBNP for diagnosing KD was 260 pg/mL, with 93% sensitivity and 88% specificity, through a comparison of 58 KD patients and 34 febrile illness patients [7].

However, previous studies did not consider the natural changes that occur in NT-proBNP levels with age. Here, we considered patient age a confounder and evaluated the different cutoff values for each disease group and the age-stratified subgroup. In each patient group, the mean NT-proBNP level decreased as age increased. In all KD patients, the cutoff value of NT-proBNP was 289 pg/mL for all age groups and 762 pg/mL for those aged < 6 months. In the incomplete KD group, the cutoff value of NT-proBNP was 261 pg/mL in all ages and 762 pg/mL in those aged < 6 months. In children < 6 months of age, applying the same NT-proBNP cutoff value for diagnosing KD regardless of age resulted in a higher sensitivity but a significantly lower specificity. Therefore, the usefulness of NT-proBNP as a diagnostic marker for KD was decreased.

We also found that the age-stratified cutoff values for patients aged 6–12 months were not significantly different from those for patients aged 12–24 months, including in terms of sensitivity and specificity. Therefore, for diagnosing KD based on NT-proBNP level, dividing the patients into three groups by age (<6 months, 6–24 months, and >24 months) and applying different cutoff values are more useful.

There are a few limitations to our study. We used a retrospective design and included a relatively small number of simple febrile illness patients as the control group. The major enrollment criterion for the control group was the performance of an NT-proBNP test. However, because of its high cost and need for extra blood samples, we did not perform an NT-proBNP test on every child with febrile illness who visited our center. Furthermore, we found relatively low sensitivity and specificity of the cutoff values compared to those in previous studies, especially in the group of patients > 24 months with incomplete KD [6, 7]. Further, large prospective investigations are required for selecting higher cutoffs of NT-proBNP as a diagnostic biomarker. Also, the NT-proBNP value has limited usefulness in patients with complete KD, which has classical diagnostic criteria. However, NT-proBNP was an independent predictor of KD despite the adjustment for CRP, platelet, and albumin levels (*P* < 0.001). The onsets of each clinical symptom differed by the diagnostic criteria. Thus, NT-proBNP can be a useful adjuvant marker for early diagnosis. Finally, the cutoff values were most useful for distinguishing incomplete KD from simple febrile illness patients < 6 months of age. However, the incidence of the disease in young infants is very low; in our center, only 25 patients were diagnosed over a 9-year period. Therefore, the clinical advantage of cutoff values obtained in this manner may be limited by the low disease incidence.

Here, we proposed cutoff values of NT-proBNP in different age groups that were useful for diagnosing complete and incomplete KD and distinguishing those conditions from simple febrile illness. For differentiating any form of KD from febrile illness, the cutoff values are 762 pg/mL (<6 months), 310 pg/mL (6–12 months), 326 pg/mL (12–24 months), and 208 pg/mL (>24 months). For diagnosing incomplete KD, the cutoff values are 762 pg/mL (<6 months), 310 pg/mL (6–12 months), 326 pg/mL (12–24 months), and 137 pg/mL (>24 months).

In conclusion, this is the first report of age-stratified measurements of NT-proBNP in patients with complete or incomplete KD and controls with simple febrile illness. Age-stratified NT-proBNP is a useful biomarker for the differential diagnosis of complete KD and incomplete KD from simple febrile illness.

## Figures and Tables

**Table 1:** Demographic characteristics and laboratory findings at diagnosis in patients with complete Kawasaki disease (KD), incomplete KD, and simple febrile illness (control).

	Complete KD		Incomplete KD		Control	
	*n*	Value	*n*	Value	*n*	Value
Age (years)	214	2.8 ± 1.8	129	2.3 ± 2.0	62	2.9 ± 2.7
Sex (boy/girl)	214	136/78	129	75/54	62	47/15
Total fever duration (days)	214	6.0 ± 1.8	129	6.3 ± 2.5	62	5.3 ± 4.5
Duration of fever until IVIg (days)	214	4.2 ± 1.9	129	4.5 ± 2.5	62	4.8 ± 3.9
White blood cell (count/mm^3^)	214	14,561 ± 4855	129	13,955 ± 5557	62	12,268 ± 4753^†^
Neutrophil (%)	213	66.3 ± 15.7	129	56.8 ± 17.3^†^	62	56.0 ± 21.0^†^
Hemoglobin (g/dL)	214	11.4 ± 1.1	129	11.2 ± 1.2	62	11.5 ± 1.3
Platelet (×10^3^/mm^3^)	214	343 ± 104	129	370 ± 145	62	279 ± 97^†,‡^
Erythrocyte sedimentation rate (mm/h)	89	64.3 ± 26.6	50	69.3 ± 32.8	21	34.7 ± 32.0^†,‡^
C-reactive protein (mg/dL)	214	8.1 ± 5.3	129	7.0 ± 5.6	62	4.8 ± 5.0^†,¶^
Aspartate aminotransferase (U/L)	212	92 ± 138	128	66 ± 104	62	85 ± 132
Alanine aminotransferase (U/L)	212	111.3 ± 170.8	128	67.8 ± 112.9^∗^	62	63.4 ± 131.8
Total bilirubin (mg/dL)	139	1.1 ± 1.4	82	0.7 ± 0.8^∗^	28	0.6 ± 0.4
Total protein (g/dL)	198	6.4 ± 0.8	117	6.5 ± 0.7	61	6.5 ± 0.6
Albumin (g/dL)	214	3.6 ± 0.6	129	3.7 ± 0.5	62	3.9 ± 0.4^†,¶^
Lactate dehydrogenase (U/L)	81	623 ± 160	53	638 ± 276	24	745 ± 258
Creatine kinase (IU/L)	181	151 ± 376	114	120 ± 248	60	272 ± 503^¶^
Creatine kinase-MB (ng/mL)	206	7.3 ± 10.5	122	4.8 ± 8.3	57	12.2 ± 11.9^†,‡^
Myoglobin (ng/mL)	130	25.4 ± 60.2	85	19.0 ± 18.6	26	74.0 ± 92.4^†,‡^
Troponin I (ng/mL)	197	0.02 ± 0.04	115	0.01 ± 0.01	45	0.01 ± 0.01
N-terminal prohormone of brain natriuretic peptide (pg/mL)	214	1785 ± 2858	129	1153 ± 1725^∗^	62	311 ± 400^†,¶^

IVIg: intravenous immunoglobulin; KD: Kawasaki disease. Data are shown as mean ± SD. ^∗^*P* < 0.05. ^†^*P* < 0.01 versus complete KD. ^¶^*P* < 0.05. ^‡^*P* < 0.01 versus incomplete KD.

**Table 2:** Laboratory findings at diagnosis in patients with complete and incomplete Kawasaki disease (KD) and simple febrile illness (control) by age.

	Complete KD		Incomplete KD		Control	
	*n*	Value	*n*	Value	*n*	Value
<6 months						
*n* (%)	21	21 (9.8)	25	25 (19.4)	8	8 (12.9)
Age (years)	21	0.4 ± 0.1	25	0.3 ± 0.1	8	0.3 ± 0.2
White blood cell (count/mm^3^)	21	13,776 ± 4361	25	16,460 ± 6412	8	13,338 ± 6342
Erythrocyte sedimentation rate (mm/h)	13	42.7 ± 21.8	7	65.6 ± 17.6	3	37.3 ± 20.5
C-reactive protein (mg/dL)	21	8.1 ± 5.9	25	8.7 ± 4.7	8	3.3 ± 2.7^‡^
N-terminal prohormone of brain natriuretic peptide (pg/mL)	21	3404 ± 3844	25	1846 ± 1811	8	669 ± 660^∗^
6–12 months						
*n* (%)	20	20 (9.3)	22	22 (17.1)	11	11 (17.7)
Age (years)	20	0.8 ± 0.2	22	0.7 ± 0.1	11	0.8 ± 0.2
White blood cell (count/mm^3^)	20	16,360 ± 3960	22	13,250 ± 6981	11	10,982 ± 3631^∗^
Erythrocyte sedimentation rate (mm/h)	10	75.3 ± 28.6	7	54.0 ± 34.9	2	2.0 ± 0.0^∗^
C-reactive protein (mg/dL)	20	7.1 ± 4.6	22	4.7 ± 5.2	11	3.7 ± 4.6
N-terminal prohormone of brain natriuretic peptide (pg/mL)	20	2241 ± 3322	22	882 ± 1381	11	301 ± 310^†^
12–24 months						
*n* (%)	39	39 (18.2)	21	21 (16.3)	12	12 (19.4)
Age (years)	39	1.6 ± 0.3	21	1.5 ± 0.3	12	1.6 ± 0.3
White blood cell (count/mm^3^)	39	14,341 ± 4611	21	12,886 ± 4688	12	12,967 ± 3444
Erythrocyte sedimentation rate (mm/h)	17	64.5 ± 23.8	7	59.6 ± 30.5	4	39.8 ± 38.2
C-reactive protein (mg/dL)	39	6.4 ± 4.2	21	4.6 ± 4.4	12	4.5 ± 4.2
N-terminal prohormone of brain natriuretic peptide (pg/mL)	39	1372 ± 1387	21	1297 ± 1951	12	398 ± 560^†,¶^
>24 months						
*n* (%)	134	134 (62.6)	61	61 (47.3)	31	31 (50.0)
Age (years)	134	3.8 ± 1.5	61	4.0 ± 1.6	31	4.9 ± 2.5
White blood cell (count/mm^3^)	134	14,479 ± 5096	61	13,551 ± 4657	31	12,177 ± 5166
Erythrocyte sedimentation rate (mm/h)	49	67.8 ± 26.0	29	76.2 ± 34.9	12	37.8 ± 34.2^∗^^,‡^
C-reactive protein (mg/dL)	134	8.8 ± 5.5	61	7.9 ± 6.0	31	5.8 ± 5.8^†^
N-terminal prohormone of brain natriuretic peptide (pg/mL)	134	1583 ± 2863	61	916 ± 1675^†^	31	188 ± 154^†,¶^

KD: Kawasaki disease. Data are shown as mean ± SD. ^∗^*P* < 0.05. ^†^*P* < 0.01 versus complete KD. ^¶^*P* < 0.05. ^‡^*P* < 0.01 versus incomplete KD.

**Table 3:** Assessment of the associations between clinical markers and Kawasaki disease using multinomial logistic regression.

Clinical marker	*χ* ^2^	*P* value
N-terminal prohormone of brain natriuretic peptide	30.52	<0.001
C-reactive protein	1.64	0.441
Platelet	30.03	<0.001
Albumin	4.79	0.091

**Table 4:** Diagnostic cutoff values of N-terminal prohormone of brain natriuretic peptide (NT-proBNP) in patients with complete, incomplete, and total (complete and incomplete) Kawasaki Disease (KD) compared to patients with simple febrile illness.

	Cutoff value of NT-proBNP(pg/mL)	Sensitivity (%)	Specificity (%)
Complete KD			
<6 months	821	71.4	75.0
6–12 months	315	85.0	81.8
12–24 months	330	74.4	75.0
>24 months	255	73.1	77.4
All ages	326	73.8	74.2
Incomplete KD			
<6 months	762	76.0	75.0
6–12 months	310	72.7	81.8
12–24 months	326	81.0	75.0
>24 months	137	70.5	54.5
All ages	261	68.8	67.2
Total KD			
<6 months	762	73.9	75.0
6–12 months	310	78.6	81.8
12–24 months	326	76.7	75.0
>24 months	208	70.8	69.7
All ages	289	71.7	71.9
